# *In vitro* Digestion and Swelling Kinetics of Thymoquinone-Loaded Pickering Emulsions Incorporated in Alginate-Chitosan Hydrogel Beads

**DOI:** 10.3389/fnut.2021.752207

**Published:** 2021-10-04

**Authors:** See Kiat Wong, Dora Lawrencia, Janarthanan Supramaniam, Bey Hing Goh, Sivakumar Manickam, Tin Wui Wong, Cheng Heng Pang, Siah Ying Tang

**Affiliations:** ^1^Chemical Engineering Discipline, School of Engineering, Monash University Malaysia, Subang Jaya, Malaysia; ^2^Biofunctional Molecule Exploratory Research Group, School of Pharmacy, Monash University Malaysia, Subang Jaya, Malaysia; ^3^College of Pharmaceutical Sciences, Zhejiang University, Hangzhou, China; ^4^Petroleum and Chemical Engineering, Faculty of Engineering, Universiti Teknologi Brunei, Bandar Seri Begawan, Brunei; ^5^Non-Destructive Biomedical and Pharmaceutical Research Centre, Smart Manufacturing Research Institute, Universiti Teknologi MARA, Puncak Alam, Malaysia; ^6^Department of Chemical and Environmental Engineering, The University of Nottingham Ningbo China, Ningbo, China; ^7^New Materials Institute, The University of Nottingham Ningbo China, Ningbo, China; ^8^Municipal Key Laboratory of Clean Energy Conversion Technologies, The University of Nottingham Ningbo China, Ningbo, China; ^9^Advanced Engineering Platform, School of Engineering, Monash University Malaysia, Subang Jaya, Malaysia; ^10^Tropical Medicine and Biology Platform, School of Science, Monash University Malaysia, Subang Jaya, Malaysia

**Keywords:** alginate, chitosan, hydrogel beads, Pickering emulsion, *in vitro* digestion

## Abstract

The present work aimed to investigate the swelling behavior, *in vitro* digestion, and release of a hydrophobic bioactive compound, thymoquinone (TQ), loaded in Pickering emulsion incorporated in alginate-chitosan hydrogel beads using a simulated gastrointestinal model. In this study, oil-in-water Pickering emulsions of uniform micron droplet sizes were formulated using 20% red palm olein and 0.5% (w/v) cellulose nanocrystals-soy protein isolate (CNC/SPI) complex followed by encapsulation within beads. FT-IR was used to characterize the bonding between the alginate, chitosan, and Pickering emulsion. 2% (w/v) alginate-1% (w/v) chitosan hydrogel beads were found to be spherical with higher stability against structural deformation. The alginate-chitosan beads displayed excellent stability in simulated gastric fluid (SGF) with a low water uptake of ~19%. The hydrogel beads demonstrated a high swelling degree (85%) with a superior water uptake capacity of ~593% during intestinal digestion in simulated intestinal fluid (SIF). After exposure to SIF, the microstructure transformation was observed, causing erosion and degradation of alginate/chitosan wall materials. The release profile of TQ up to 83% was achieved in intestinal digestion, and the release behavior was dominated by diffusion via the bead swelling process. These results provided useful insight into the design of food-grade colloidal delivery systems using protein-polysaccharide complex-stabilized Pickering emulsions incorporated in alginate-chitosan hydrogel beads.

## Introduction

Thymoquinone (TQ), a major essential oil component of *Nigella sativa* seeds, has attracted significant attention in recent years to be used as an alternative to chemical drugs. TQ has been known to possess therapeutic benefits such as anti-oxidant, anti-inflammatory, and anti-cancer with minimal adverse effects and no severe toxicity (1, 2). Furthermore, several studies showed that TQ could induce apoptosis in human colorectal cancer cells by abrogate the stress response pathway sensor CHEK1 (3) and inhibit the proliferation of human colon cancer cells by increasing the phosphorylation states of the mitogen-activated protein kinases (4, 5). These findings suggest that TQ could be a critical component in formulating nutraceutical food products for colon cancer prevention. However, the application of TQ in food systems has been limited because of its high hydrophobicity due to the low dissolution profile when delivered orally. Hence, it exhibits low bioavailability at the target-diseased site (6). In addition, researchers found that TQ also suffers from chemical decomposition and enzymatic degradation in the gastrointestinal tract, which further limits the oral administration of TQ (7). These limitations could be effectively overcome by physically entrapping TQ in delivery colloid carrier systems to enhance its bioavailability and therapeutic ability.

Natural resources such as protein, lipid and polysaccharides have been used as environmentally sustainable biomaterials for the active coating of the bioactive agent (8). One of the prominent carriers for encapsulation, the alginate hydrogel beads, have been investigated extensively over the last decade because of their unique properties such as good aqueous solubility, biocompatibility, biodegradability, and non-toxicity. These characteristics make them helpful in encapsulating food ingredients, drugs, or natural extracts in the food industry and drug delivery system (9–12). The system can encapsulate small and large molecules at high efficiencies (13). It can readily form a three-dimensional gel-like structure, known as the egg-box model, when crosslinking with divalent cations such as Ca^2+^ (14). However, mainly hydrophilic molecules are selected as encapsulants resulting from many free hydroxyl and carboxyl groups along the alginate backbone (15). To encapsulate hydrophobic molecules, chemical modification is required by introducing hydrophobic groups such as lipid into the hydrogel beads matrix.

Emulsions are a mixture of two or more immiscible liquids, often stabilized with surfactants or surface-active particles. They have been widely used in food products due to their excellent stability and high nutritional value (16, 17). Due to the lipophilic property of TQ, an oil-in-water (O/W) emulsion is a perfect carrier system for the encapsulation of TQ. In particular, Pickering emulsions (PE) are emulsions stabilized with solid particles in place of surfactants. PE has shown numerous advantages of robust stabilization against coalescence with enhanced phase separation due to a dense layer of solid particles adsorbed irreversibly around the emulsion droplets (18). Liu and Tang confirmed that soy protein nanoparticles (0.5–6.0%, w/w) could formulate Pickering emulsions with properties tailored by changing the concentration and emulsification process (19). Yi et al. found that the combined use of soy protein isolate (20 mg/mL) and gallic acid (0.5–1.5 mg/mL) as the Pickering stabilizers contributed to great stability, excellent anti-oxidant, and antimicrobial abilities (20). Most recently, Wong et al. demonstrated the preparation of highly stable yet uniform red palm olein-in-water Pickering emulsions using cellulose nanocrystals-soy protein isolate (1.0%:1.0%, w/w) nanoconjugates as the food-based stabilizer (21).

However, protein particles-stabilized PE is often susceptible to changes in pH, especially during gastric digestion. Hence, by dissolving TQ in the oil phase of PE, followed by encapsulating the TQ-loaded PE within the alginate hydrogel matrix, bioactive compounds can be protected from the harsh external environment by improving the bioavailability of orally administered hydrophobic molecules (22, 23). Several studies have demonstrated the application of hydrogel in the immobilization of Pickering emulsion. For instance, Xiao et al. fabricated curcumin-loaded Pickering emulsion alginate hydrogel with improved processing stability and controlled digestion profile (24). In addition, Yan et al. reported the encapsulation of alfacalcidol in alginate beads using Pickering emulsion as a template, and the composite beads exhibited sustained release performance (11). In another study, Lim et al. successfully prepared chitosan-stabilized Pickering emulsions with immobilization efficiency exceeding 99% using calcium-alginate hydrogel (25).

To improve the stability and rigidity of alginate hydrogel beads, chitosan, a deacetylated product of chitin, which is the second most abundant polysaccharide after cellulose, can be employed as a supporting polymer. Chitosan has excellent biocompatibility, biodegradability, and mucoadhesive properties, and it is the only polysaccharide exhibiting cationic character, making it a suitable material in the food and nutraceutical industry (26, 27). When used together in forming hydrogel beads, the cationic amine group (${\text{-NH}}_{3}^{+}$) of chitosan could bind to the carboxylate group (-COO^−^) of alginate, forming crosslinked alginate-chitosan complexes, providing an enhanced protective coating for the encapsulated moieties (28, 29). It is also reported that the stability of the protein-based delivery system could be benefited from the chitosan coating, where proteins hydrolysis can be decreased during gastrointestinal digestion (30).

**Figure 1:**
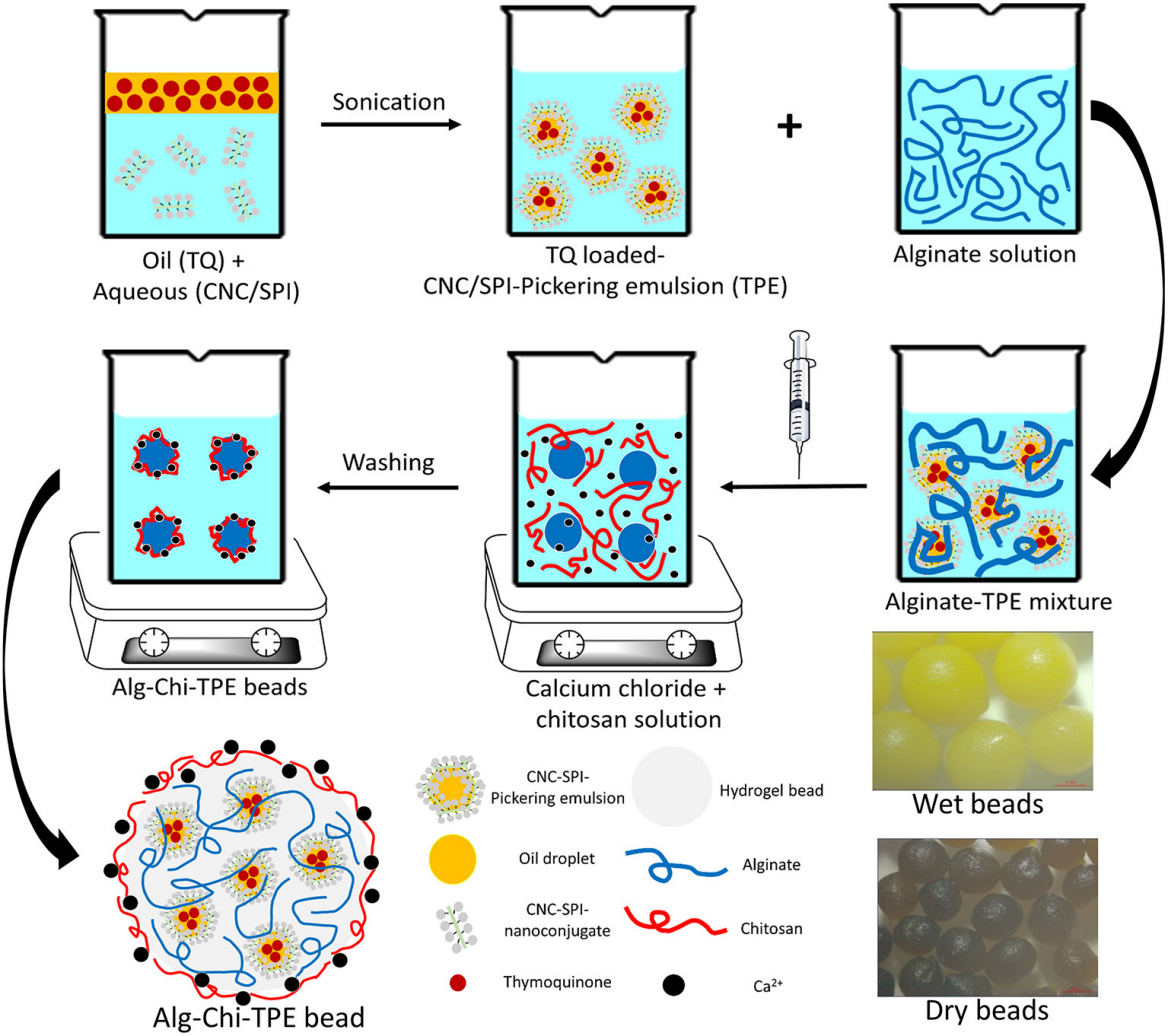
The schematic illustration of the preparation of thymoquinone loaded alginate-chitosan beads by immobilized CNC/SPI-stabilized Pickering emulsion.

To date, the encapsulation of Pickering emulsions in alginate-chitosan beads with a hydrophobic bioactive component is rarely reported, especially when using a polysaccharide-protein complex as the Pickering emulsifier. This study combined the highly efficient encapsulating performance of alginate-chitosan (Alg-Chi) hydrogel beads with cellulose nanocrystals-soy protein isolate (CNC/SPI)-stabilized Pickering emulsion to develop a new delivery formulation for the loading of the hydrophobic bioactive model, TQ. The CNC/SPI-stabilized Pickering emulsion (TPE) was prepared by ultrasonication using a probe based on a previous study (21) and an ionic gelation method to fabricate Alg-Chi-TPE hydrogel beads, as shown in Figure 1. The morphology, encapsulation efficiency, *in vitro* digestion and release performance of Alg-Chi-TPE hydrogel beads were investigated. The findings of the current work could contribute to the practical applications of polysaccharide-protein-based Pickering emulsions in nutraceutical and functional food products.

## Materials and Methods

### Materials

Cellulose nanocrystals (CNC) (freeze-dried) were procured from the University of Maine, United States. Soy protein isolate (SPI) was provided by Shandong Wonderful Industrial Group Co., Ltd. (Shandong, China). Sodium alginate, calcium chloride and low molecular weight chitosan (50–190 kDa) were purchased from Sigma Chemicals Co. (St. Louis, MO, USA). Red palm superolein (275 ppm β-carotene, melting point 19°C) was acquired from Sime Darby Jomalina Sdn. Bhd., Malaysia. Ultrapure water (18.2 MΩ cm^−1^) was obtained from the Milli-Q^®^ Plus apparatus (Millipore, Billerica, USA) and was used in all the experiments. All other chemicals and reagents used were of analytical grade.

### Preparation of Thymoquinone-Loaded Oil-in-Water (O/W) Pickering Emulsion (TPE)

CNC/SPI complex was used as the solid stabilizer in forming O/W Pickering emulsion with red palm olein (deep orange color) as the oil phase and deionized water as the continuous aqueous phase. Thymoquinone (TQ) was firstly dissolved in the oil phase with magnetic stirring at 500 rpm at room temperature for 1 h. Next, emulsions with a fixed oil content were prepared by mixing 20% (v/v) palm olein with aqueous suspensions of 0.5% (w/v) CNC/SPI complex at pH 7, adjusted by 1 M HCl and 1 M NaOH. Subsequently, the mixture was emulsified for 5 min using the ultrasonic horn at 60 W, and the emulsion was stored in glass vials for further usage. The measured average droplet size of formulated TPE was found to be ~23.5 ± 2.2 μm with a zeta potential of 34.3 ± 1.4 mV (21). Pickering emulsions without TQ was prepared similarly as control.

### Preparation of Alginate-Chitosan (Alg-Chi) Beads Immobilized TPE

According to a previously described method (31), alginate-chitosan beads immobilized Pickering emulsions (Alg-Chi-TPE) were produced using the ionic gelation method with modification. Firstly, sodium alginate was dissolved in ultrapure water to give sodium alginate solutions of different concentrations (1%, 2%, 3%, 4% w/v). These sodium alginate solutions were degassed for 30min to discharge air bubbles before being utilized. Next, freshly prepared Pickering emulsions were added in alginate solutions at a 1:1 (v/v) ratio and mixed homogeneously under magnetic stirring for 10 min to produce Pickering emulsion-alginate solutions with final alginate concentrations (0.5%, 1%, 1.5%, 2% w/v). Then, Pickering emulsion-alginate solution was added dropwise into a mixed solution of 2% (w/v) calcium chloride solution in ultrapure water and 1% (w/v) chitosan solution in 1% (v/v) acetic acid solution under gentle stirring through a 21-gauge needle syringe. The formed beads were hardened in the mixed solution for 30min before collection and washed with ultrapure water. The mixed and washing solutions were kept to measure the encapsulation efficiency of thymoquinone in the Pickering emulsion-filled alginate-chitosan beads.

### Characterization of Alginate-Chitosan (Alg-Chi) Beads Immobilized TPE

#### Shape and Size of Hydrogel Beads

The shape and average size of freshly formed Alg-Chi-TPE hydrogel beads and dried Alg-Chi-TPE hydrogel beads were investigated using a Stemi microscope (Stemi2000, Zeiss, Germany). The size of the beads was measured using the ZEN2012 Blue software and was averaged based on eight replicates. The shrinking ratio of freshly prepared and dried alginate-chitosan beads was calculated using the following Equation (1).


(1) $$\begin{array}{l} Shrinking\;ratio\;\left(\%\right)=\frac{S_{f}-\;S_{d}}{S_{f}}x\;100\% \end{array}$$

Where S_f_ is the average size of freshly prepared beads and S_d_ is the average size of dried beads.

#### FT-IR Spectroscopy

Alg-Chi-TPE hydrogel beads were crushed using mortar and pestle before analysis. The FTIR spectra over the wavelength range of 400–4,000 cm^−1^ were recorded using the attenuated total reflectance Fourier transform infrared (ATR-FT-IR) spectrometer (Nicolet iS10, Thermo Fisher Scientific, USA).

#### Measurement of Encapsulation Efficiency (EE)

According to the previously described method for O/W emulsions (32), the encapsulation efficiency was analyzed by centrifugation with modification. In brief, 1 mL of O/W emulsion was added dropwise into a mixture of isopropanol and hexane (1:1 v/v, 10 mL) and subjected to vortex for 20 s to break the emulsion. Then, the mixture was centrifuged at 4,500 rpm for 10 min. The amount of thymoquinone in the supernatant was measured at the wavelength of 290 nm by a UV-Vis spectrophotometer (Genesys 10s UV, Thermo Fisher Scientific, USA). O/W emulsion without thymoquinone was prepared similarly and used as a blank. The encapsulation efficiency of thymoquinone in O/W emulsions (EE_e_) was calculated based on a standard calibrated curve of thymoquinone based on the following Equation (2).


2 $$\begin{array}{r} EE_{e}\;\left(\%\right)=\left(\frac{Amount\;of\;thymoquinone\;in\;the\;supernatant}{Amount\;of\;total\;thymoquinone}\right)\\ \;\times \;100\% \end{array}$$

According to the previously described method for O/W emulsion-filled alginate-chitosan (Alg-Chi-TPE) hydrogel beads, the encapsulation efficiency was calculated by measuring the leakage during the crosslinking process (30). After the hardening process of beads, the absorbance of the mixed solution (CaCl_2_ and chitosan) and the washing solution was measured at the wavelength of 290 nm by a UV-Vis spectrophotometer (Genesys 10s UV, Thermo Fisher Scientific, USA). The encapsulation efficiency of thymoquinone in Alg-Chi-TPE hydrogel beads (EE_b_) was calculated using the following Equation (3).


(3) $$\begin{array}{r} EE_{b}\;\left(\%\right)=\left(1-\frac{Amount\;of\;thymoquinone\;in\;the\;mixed\;solution}{Amount\;of\;total\;thymoquinone}\right)\\ \;\times \;100\% \end{array}$$

### *In vitro* Digestion

Simulated digestion of Alg-Chi-TPE hydrogel beads in gastric and intestinal phases was performed according to a previously described method (32) with modification. Simulated gastric fluid (SGF) and simulated intestinal fluid (SIF) were prepared as described below. SGF was prepared by dissolving 2 g of NaCl, 3.2 g of pepsin and 7 ml of 12 M HCl in 1 L of ultrapure water. SIF was prepared by adding pancreatic lipase (4 mg/mL), bile salt (4.3 mg/mL), and 0.6 mM CaCl_2_ in phosphate buffer solution (pH 7.5). The mixture was stirred until homogenized for 1 h before use. Pepsin is responsible for breaking down protein during gastric digestion, while pancreatic lipase is important in the dietary triacylglycerol breakdown during intestinal digestion.

#### Water Uptake Study

The water uptake of the Alg-Chi-TPE hydrogel beads was performed in two different digestive media: SGF and SIF. Accurately weighed beads were immersed in 20 mL of respective medium in a sealed conical flask and placed in an incubator shaker (100 rpm) at 37°C. The beads were separated from the medium at specific time intervals, wiped gently with filter paper and weighed. The weight change of the beads to time was determined using the following Equation (4).


(4) $$\begin{array}{l} Water\;uptake\;\left(\%\right)=\frac{W_{s}-W_{i}}{W_{i}}\;x\;100\% \end{array}$$

Where W_s_ is the weight of the beads in swollen state and W_i_ is the initial weight of the untreated beads.

#### Swelling and Erosion Study

The swelling and erosion of the Alg-Chi-TPE hydrogel beads were performed in two different digestive media: SGF and SIF. Beads were immersed in 20 mL of respective medium in a sealed conical flask and placed in an incubator shaker (100 rpm) at 37°C. The beads were removed from the medium at specific time intervals, wiped gently with filter paper, and the diameter was measured. The swelling degree and erosion degree of the beads to time were determined using the following Equations (5) and (6), respectively.


(5) $$\begin{array}{l} Swelling\left(\%\right)=\frac{D_{s}-D_{i}}{D_{i}}\;x\;100\% \end{array}$$

(6) $$\begin{array}{l} Erosion\;\left(\%\right)=\frac{W_{i}-W_{e}}{W_{i}}\;x\;100\% \end{array}$$

Where D_s_ is the diameter of the beads in the swollen state, D_i_ is the initial diameter of the untreated beads, W_e_ is the weight of the dried beads in the swollen state, and W_i_ is the initial weight of the untreated beads.

#### Microstructures of Beads After Digestion

The microstructures of Alg-Chi-TPE hydrogel beads after gastric digestion and intestinal digestion were observed through a variable pressure scanning electron microscope (VP-SEM, Hitachi s3400N-II, Japan) and an upright fluorescent microscope (Nikon Eclipse 90i, Nikon Instrument Inc., USA). For VP-SEM preparation, the samples were air-dried, sputter-coated with gold under a vacuum before analysis. The samples were observed at an accelerating voltage of 10 kV. For the fluorescent microscope, the lipid phase of the emulsions was stained with Nile red (0.01% w/v) during the preparation process. A bead was placed on a microscopic slide and gently covered with a coverslip. The edge of beads was observed.

#### Release Study of Thymoquinone During Digestion

The release of thymoquinone (TQ) from Alg-Chi-TPE hydrogel beads in gastric digestion and intestinal digestion were evaluated according to the method described in section Measurement of Encapsulation Efficiency (EE). In brief, the hydrogel beads were immersed in 20 mL of respective medium in a sealed conical flask and placed in an incubator shaker (100 rpm) at 37°C. The hydrogel beads were treated with SGF for 120 min followed by SIF for another 240 min. Then, at specific time intervals, 2 mL of the medium was withdrawn from the conical flask and topped up with the fresh medium. The release profiles of TQ were fitted to the Peppas model by the following Equation (7) (33).


(7) $$\begin{array}{l} \frac{Mt}{Mi}=\;kt^{n} \end{array}$$

Where Mt/Mi is the cumulative release ratio at time t, k is the kinetic constant, and n is the diffusional exponent.

## Results and Discussion

### Fabrication and Characterization of Alginate-Chitosan Beads Immobilized Thymoquinone-Loaded Pickering Emulsion

**Figure 2:**
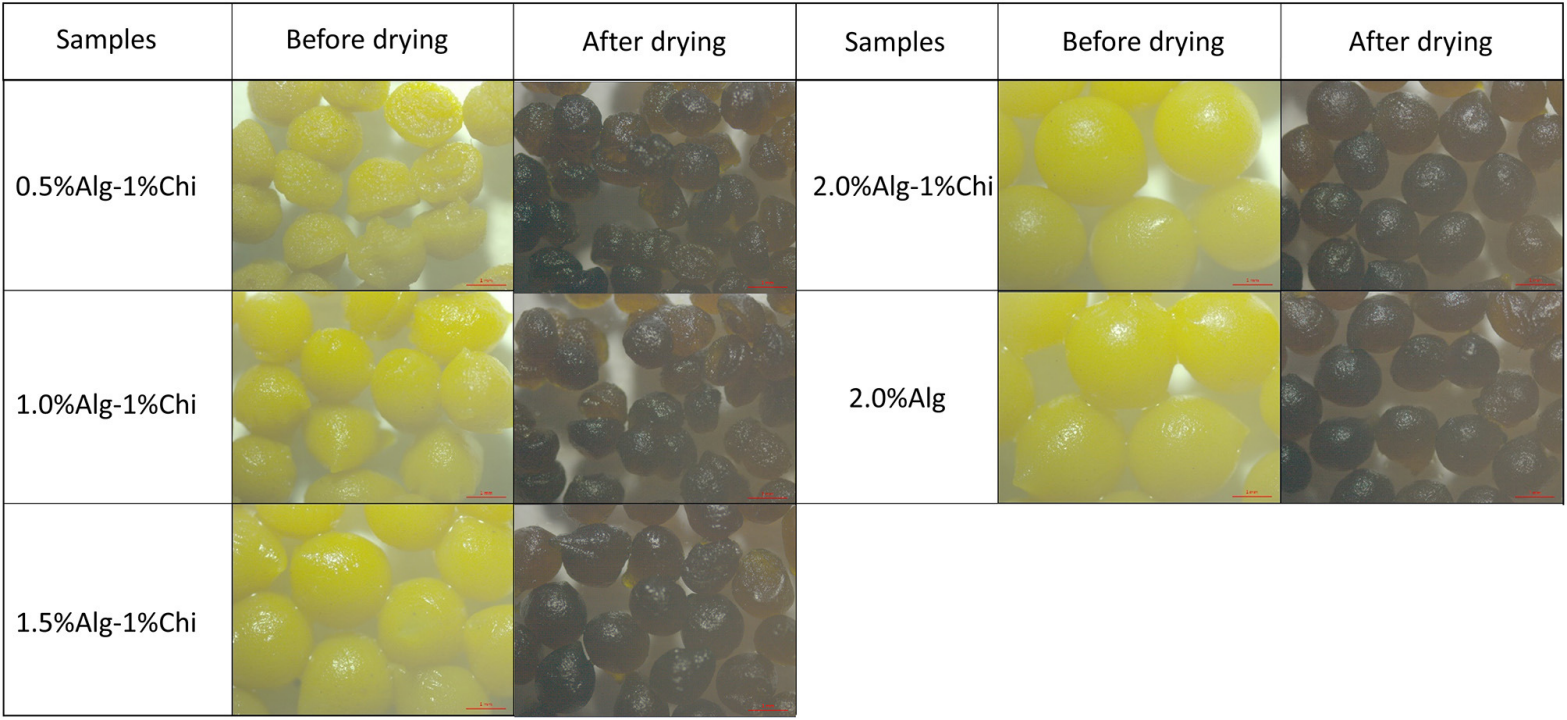
Digital images of wet and dry alginate-chitosan hydrogel beads immobilized Pickering emulsion obtained with different alginate concentrations.

Thymoquinone (TQ) was first dissolved in the red palm olein phase before the emulsification process. Next, the cellulose nanocrystals-soy protein isolate (CNC/SPI) complex was used as the Pickering stabilizer and dispersed in the aqueous phase. The alginate-chitosan (Alg-Chi) hydrogel beads were then prepared by external gelation method through dripping a mixture of TQ-loaded CNC/SPI-stabilized Pickering emulsion (TPE) with different alginate concentrations into a pre-mixed CaCl_2_/chitosan solution (Figure 1). The digital images of the wet and dry Alg-Chi-TPE hydrogel beads are shown in Figure 2. Both wet and dry beads exhibited uniformly spherical shapes when 2% (w/v) alginate was used. However, when lower alginate concentrations were used, the shape of the beads was deformed, resulting in rough and collapsed surface morphology. This deformation is inevitable when water was evaporated from the wet hydrogel beads during the drying process, causing volume shrinkage of hydrogel beads (34).

Table 1 summarizes the percentage of weight change after the drying process. It could be observed that 2% (w/v) alginate beads exhibit the highest difference in shrinking ratio compared to the rest due to the higher concentration available to form larger wet beads with more water content. Generally, increasing the alginate ratio in beads formulation causes the beads' amplification and weight change. However, hydrogel beads obtained with 2.0% (w/v) alginate concentration displayed the least structural deformation after the drying process and demonstrated better stability than the other hydrogel beads. It is worth noting that the colors of the beads became darker when the beads shrink, which could be due to the increase in the concentration of immobilized TPE within the beads after the water has evaporated.

**Table 1:** The mean sizes and the shrinking ratio of Alg-Chi hydrogel beads were determined by ZEN2012 Blue software.

	**Wet (mm)**	**Dry (mm)**	**Shrinking ratio (%)**
0.5% Alg-1.0% Chi	1.598 ± 0.106^a^	1.138 ± 0.077^a^	28.65 ± 0.10
1.0% Alg-1.0% Chi	1.987 ± 0.118^b^	1.383 ± 0.052^b^	31.84 ± 1.47
1.5% Alg-1.0% Chi	2.339 ± 0.155^c^	1.554 ± 0.068^c^	34.96 ± 1.39
2% Alg-1.0% Chi	2.699 ± 0.097^d^	1.613 ± 0.061^c, d^	40.13 ± 0.11
2% Alg	2.651 ± 0.102^d, e^	1.583 ± 0.044^c, d, e^	40.94 ± 0.63

*Values expressed as the mean ± standard deviation. Different letters in the same column indicate significant differences (p < 0.05)*.

**Figure 3:**
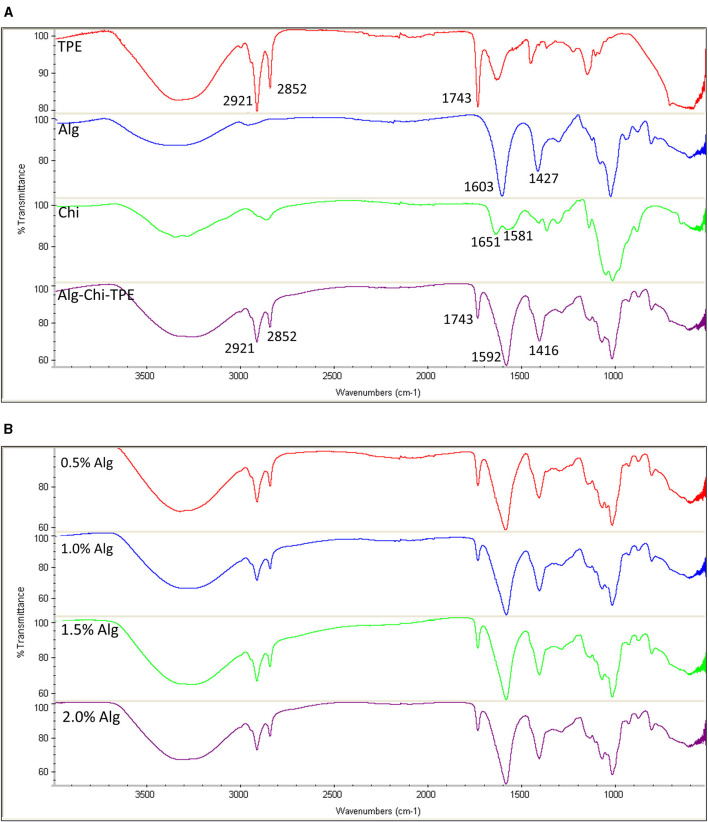
FTIR spectra of **(A)** TPE, Alg, Chi, and Alg-Chi-TPE beads **(B)** different alginate concentrations (0.5–2.0% w/v).

FTIR spectra of thymoquinone-loaded Pickering emulsion (TPE), Alg, Alg-TPE, Chi, and Alg-Chi-TPE hydrogel beads are shown in Figure 3A. The typical bands for the fatty acid hydrocarbon chains of palm olein in TPE could be observed at 2,921, 2,852, and 1,743 cm^−1^ for the asymmetric and symmetric stretching vibrations of C-H (-CH2) and carbonyl group (C=O) from ester (35). The FTIR spectrum of Alg-Chi-TPE beads revealed similar profiles as the TPE with the additional strong peaks at 1,592 and 1,416 cm^−1^, attributed to the asymmetric and symmetric stretching vibrations of the -CO bond in the carboxylate (-COO^−^) group of alginate (36). The shift observed from 1,603 to 1,592 cm^−1^ and 1,427 to 1,416 cm^−1^ in the spectra could be due to ion-induced alginate gelation by Ca^2+^ and cationic chitosan. The similarities of the palm olein bands revealed no interaction between the Pickering emulsion and wall materials, indicating that the Pickering emulsion is physically entrapped within the Alg-Chi beads system. Chitosan spectrum shows characteristic bands at 1,651 and 1,583 cm^−1^ due to the C=O vibration of the acetylated units (-CONH_2_ groups) (37). The C=O vibration in the Alg-Chi-TPE spectrum shifted to a lower wavelength (1,651 to 1,592 cm^−1^), denoting the electrostatic interaction of chitosan with alginate in the hydrogel beads. On the other hand, the FTIR spectra (Figure 3B) of Alg-Chi-TPE hydrogel beads with different Alg concentrations displayed similar profiles suggesting that Alg concentration does not affect the physicochemical interactions between the immobilized TPE and the wall materials.

According to Equation (2), the encapsulation efficiency (EE_e_) of thymoquinone (TQ) in the freshly prepared Pickering emulsion system was determined to be >99%. This is reasonable since TQ first fully dissolves in the oil phase before turning into an O/W emulsion. Alternatively, based on the hydrogel shells formed by external gelation that immobilized the O/W emulsion loaded with TQ, the TQ's encapsulation efficiency (EE_b_) according to Equation (3) of the Alg-Chi-TPE beads was determined to be 89.18 ± 3.63%, revealing an excellent loading efficiency using hydrogel beads. A decrease in the encapsulation efficiency could be due to the release of TQ during the ionic gelation and the hardening process.

### Effects of Digestion on Alginate-Chitosan Beads Immobilized Thymoquinone-Loaded Pickering Emulsions

**Figure 4:**
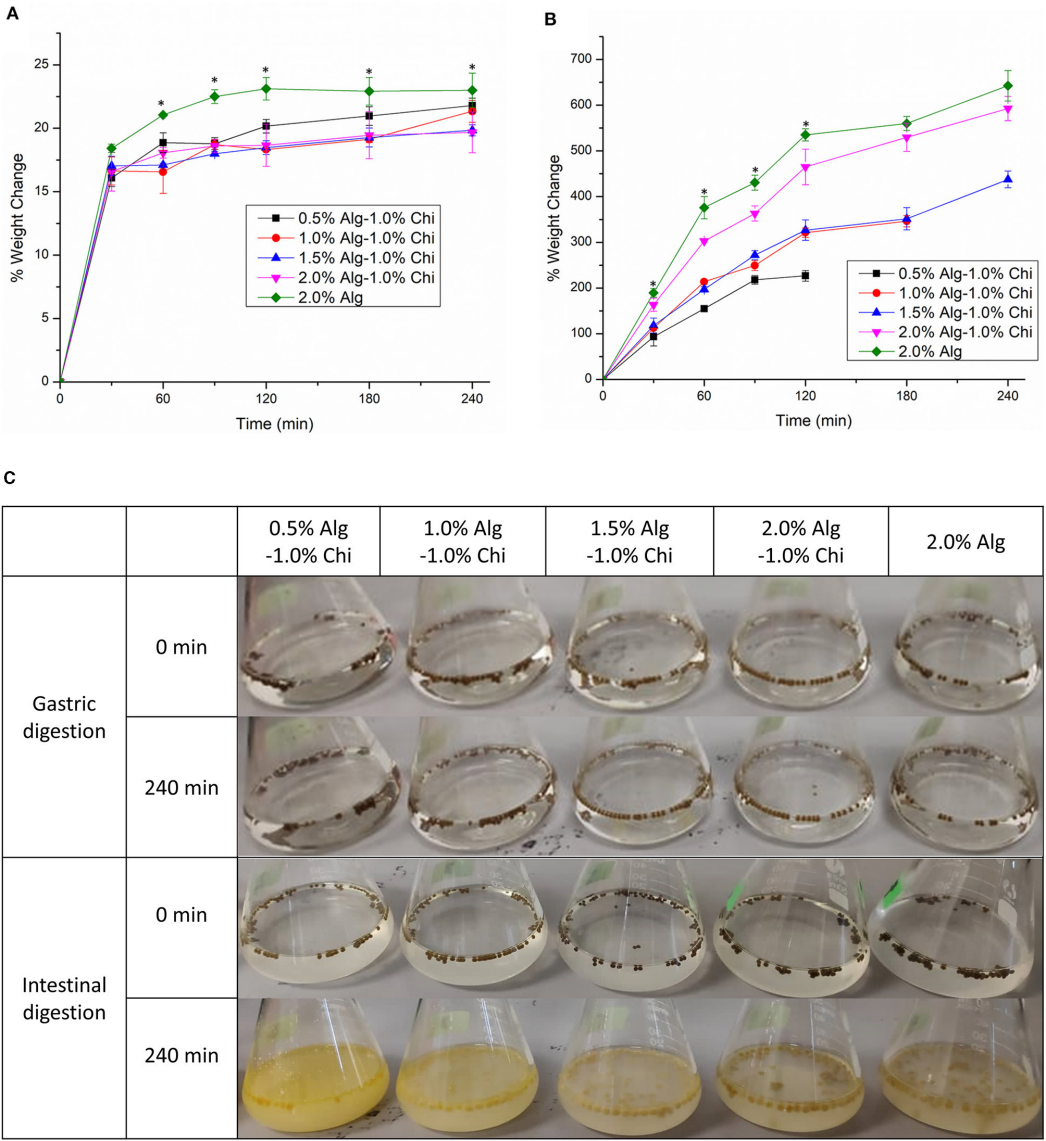
The water uptake of alginate-chitosan hydrogel beads immobilized Pickering emulsion obtained with different alginate concentrations during **(A)** gastric digestion **(B)** intestinal digestion. **(C)** The visual appearance of beads before and after gastric and intestinal digestion. Error bars represent the standard deviation of three replicates. ^*^Marks significant differences (*p* < 0.05). A comparison was made between 2.0% Alg and 2.0–1.0% Chi.

Figure 4 illustrates the water uptake of Alg-Chi-TPE hydrogel beads with different Alg concentrations in simulated gastric fluid (SGF) and simulated intestinal fluid (SIF). All hydrogel beads exhibited a weight increase of ~15% after 30min of gastric digestion. The increase in weight can be justified where the void regions within the hydrogel beads get filled up by water due to osmotic pressure asserting on the hydrogel beads. The water uptake ratio of all the beads continued to rise to a maximum of 23% for beads formed with 2.0% (w/v) Alg. The water uptake is minimal at the low pH of gastric fluid because alginate precipitates to form alginic molecules in the form of aggregates linked by hydrogen bonding leading to higher stability (38). When the hydrogel beads were introduced to the gastric digestive fluid, denser alginate structures were believed to be due to the weakened electrostatic repulsion among the alginate molecules. The pH of the SGF was maintained at pH 1.5, and the pK_a_ of alginate was at about 3.5, causing the alginate to reduce its negative charges (39).

The water uptake of beads was accelerated during intestinal digestion, which is ideal for digesting oils. Hydrogel beads obtained with 0.5% and 1.0% (w/v) alginate were disintegrated and deformed, where the weight measurement of the swollen beads could not proceed (Figure 4B). Generally, the water uptake ability of all beads during intestinal digestion improved with increasing alginate concentration. The water uptake ratios of 2.0% (w/v) alginate and 2.0% (w/v) alginate-chitosan beads were significantly different at 120 min (*p* < 0.05) and continued to achieve 643 and 593%, respectively at 240 min, demonstrating substantial intestinal digestion of hydrogel beads in SIF. The destabilization of the alginate polymer network at intestinal pH is due to the ion exchange between Na^+^ ions in the intestinal digestion fluid and Ca^2+^ ions in the hydrogel beads (40). The calcium ions dissociate and form calcium phosphate salts which no longer crosslink with the alginate matrix, leading to the structural degradation of the hydrogel matrix. On the other hand, the ionization of alginate at intestinal pH produces electrostatic repulsion forces between alginate chains, increasing weight gain (32).

Table 2 shows the swelling and erosion degrees of Alg-TPE and Alg-Chi-TPE hydrogel beads after 120 min of gastric digestion and up to 240 min of intestinal digestion. The swelling degree measures the diameter of the swollen beads after treatment. The swelling degree for both hydrogel beads reduced after 120 min of gastric digestion, demonstrating the shrinking of beads. The shrinking occurs due to the dissociation of Ca^2+^ ions at low pH, the COO^−^ groups become protonated, resulting in the formation of hydrogen bonds within the alginate chains (41). In contrast, the swelling degree increased tremendously to around 100 and 85% after 240 min of intestinal digestion for Alg-TPE and Alg-Chi-TPE hydrogel beads, respectively. This observation is in line with other studies where alginate-based hydrogel beads shrunk during gastric digestion and swell during intestinal digestion, possibly due to the changes in electrostatic forces of the wall matrix at different pH (31, 40). It is worth noting that the hydrogel beads with chitosan illustrated a lower swelling degree than the hydrogel beads without chitosan. The addition of chitosan onto alginate leads to the formation of a more entangled system developed by the blending of both polymers forming polyelectrolyte complexes between the amino groups of chitosan and carboxylate groups of alginate (31). These factors improved the stability of Alg-Chi-TPE hydrogel beads and exhibited increased resistance to osmotic pressure.

**Table 2:** The swelling and erosion degrees of alginate hydrogel beads and alginate-chitosan hydrogel beads during gastric digestion and intestinal digestion.

	**2.0% Alg**		**2.0% Alg-1.0% Chi**	
**Time (min)**	**Swelling (%)**	**Erosion (%)**	**Swelling (%)**	**Erosion (%)**
SGF-120	−3.38 ± 3.76^a^	8.24 ± 0.48^c^	−9.12 ± 3.32^b^	6.56 ± 0.84^d^
SIF-30	27.51 ± 4.92^a^	4.49 ± 0.45^c^	18.64 ± 4.03^b^	3.83 ± 1.04^d^
SIF-60	56.27 ± 6.74^a^	6.23 ± 0.61^c^	48.55 ± 5.29^a^	4.66 ± 0.56^d^
SIF-120	75.06 ± 7.92^a^	7.81 ± 0.23^c^	54.75 ± 5.47^b^	5.76 ± 0.79^d^
SIF-180	90.13 ± 7.02^a^	12.18 ± 0.90^c^	68.72 ± 6.82^b^	7.66 ± 0.85^d^
SIF-240	100.23 ± 8.83^a^	15.13 ± 0.77^c^	85.22 ± 6.01^b^	13.83 ± 1.01^c^

*Values expressed as the mean ± standard deviation. Different letters in the same row indicate significant differences (p < 0.05). A comparison was made between 2.0% Alg and 2.0–1.0% Chi. SGF—gastric digestion, and SIF—intestinal digestion*.

On the other hand, the erosion degree measures the weight loss of the dried hydrogel beads after treatment. From Table 2, the erosion degrees for both Alg-TPE and Alg-Chi-TPE hydrogel beads after gastric digestion were 8.24 and 6.56%, respectively (*p* < 0.05). A significant reduction in the weight of hydrogel beads can be related to the syneresis effect in an acidic environment where shrinkage is favored (42). Nevertheless, the erosion degree of the hydrogel beads in an alkaline environment begins to rise with time, with the maximum erosion degree of 13.83% at 240 min for hydrogel beads coated with chitosan and 15.13% for hydrogel beads without chitosan. The erosion degree for hydrogel beads coated with chitosan was lower than hydrogel beads without chitosan, demonstrating enhanced stability of the hydrogel beads when chitosan was introduced. As the treatment continues, the degradation, and dissolution of the bead matrix were enhanced over time. As a result, the final weight of the dried and treated hydrogel beads became lower, displaying time-dependent erosion properties.

**Figure 5:**
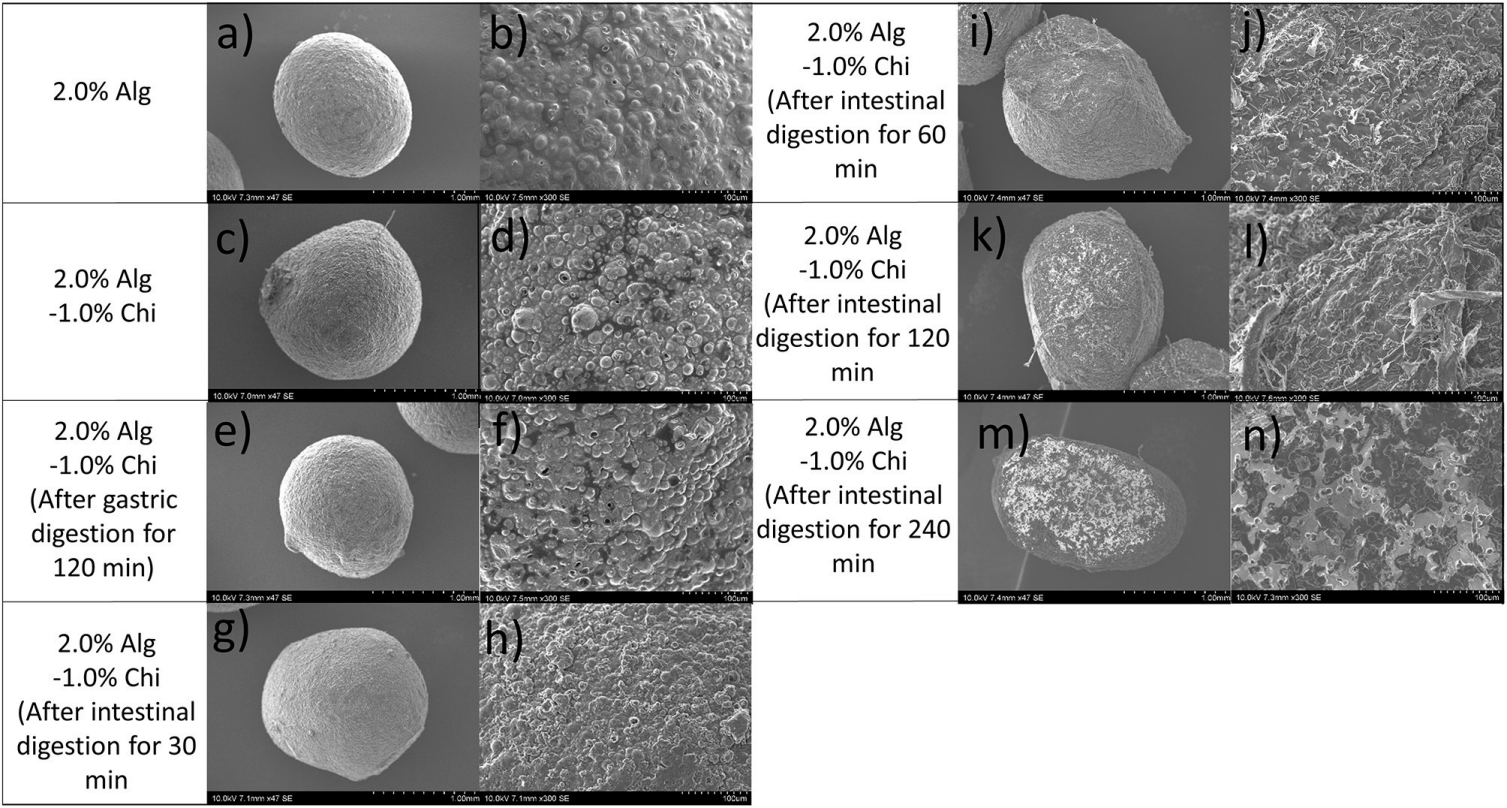
SEM images of **(a,b)** 2.0% Alg hydrogel beads, **(c,d)** 2.0% Alg-1.0% Chi hydrogel beads, **(e,f)** 2.0% Alg- 1.0% Chi hydrogel beads after 120 min gastric digestion, **(g–n)** 2.0%- 1.0% Chi hydrogel beads after 30-240 min intestinal digestion at ×47 magnification (left) and ×300 magnification (right).

The hydrogel beads prepared by 2.0% alginate were used to study the effects of digestion on the microstructures and bioactive release profile based on the swelling performance reported above. Scanning electron micrographs of dry alginate hydrogel (Alg-TPE) beads, chitosan-coated alginate (Alg-Chi-TPE) beads and Alg-Chi-TPE beads after specific digestion in SGF or SIF are illustrated in Figure 5. The Alg-TPE and Alg-Chi-TPE hydrogel beads exhibited spherical shape after air drying, and a detailed examination of the surface structure revealed a rough and folded appearance. There was not much difference between alginate and alginate-chitosan beads in terms of surface structure. However, a closer observation on SEM images with higher magnification (x300) illustrated the presence of more rough surfaces with irregular dents on the crosslinked alginate-chitosan hydrogel beads (Figures 5b,d). This could be attributed to the effect of chitosan polymer coating onto the surface of the alginate matrix, creating patchy-like textures associated with a shielding effect by the insoluble chitosan layer. The results can be correlated to the lower swelling profile of chitosan-coated alginate beads than the uncoated beads [swelling degree of 85 vs. 100% at 2.0% (w/v) alginate concentration].

As shown in Figures 5d,f, there is no significant variation in the microstructure of beads after gastric digestion with similar roughness and compact surfaces. This observation agrees with the swelling behaviors of hydrogel beads, where alginate displayed excellent stability in a medium of low pH. The stability of the alginate-chitosan beads or capsules depends strongly on the differences in their assembly (43) and the amount of chitosan bound to the capsules (44). The present study employed a one-step preparative procedure by dropping the emulsion-alginate mixture into a chitosan solution containing calcium chloride. Hydrogel bead formation was achieved by the ionic gelation effect, and chitosan formed the outer layer of the beads. An earlier study reported that the chitosan molecules could diffuse into the alginate matrix, creating a 3D hydrogel network interconnected by alginate molecules, chitosan polymer bridges, and cationic calcium ions (45). The SEM micrographs revealed minimal changes in the surface morphology of alginate-chitosan beads upon 120 min exposure to SGF. A similar observation was also made in a previous work conducted by Li et al. (46). In addition, Chew et al. concluded that the chitosan-alginate coacervated beads appear to resist in an acidic medium where the structure remains intact because of the ionic bonds of calcium-alginate-chitosan complexation through electrostatic interactions (47).

**Figure 6:**
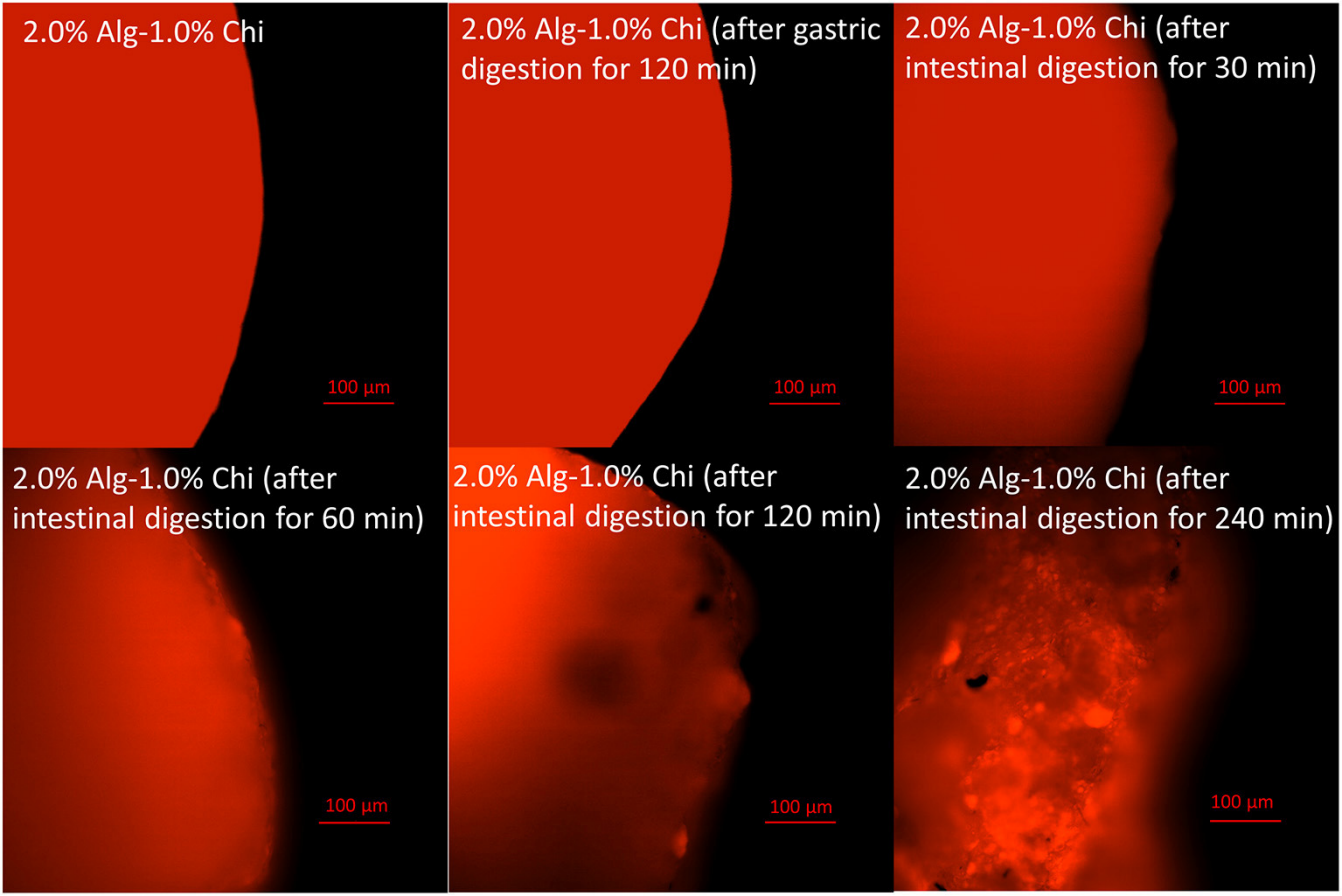
Fluorescent images of alginate-chitosan hydrogel beads immobilized Pickering emulsion surface after gastric and intestinal digestion (scale bar, 100 μm).

The micrographs of Alg-Chi-TPE hydrogel beads at a different time point of intestinal digestion are illustrated in Figures 5g–n. After 30min of intestinal digestion, the microstructure of hydrogel beads displayed a smoother surface, indicating the degradation of the hydrogel bead wall materials (Figures 5g,h). The compact structure gradually transforms into a heterogeneous structure with disorderly folded and significant dents when the intestinal digestion time increased to 120 min. The change in the microstructures of hydrogel beads could be due to the swelling process, which coincides with the erosion and dissolution of swollen beads (32). A slight deformation on the morphology of hydrogel beads (spherical to oval) after 60 min of intestinal digestion (Figures 5i,j) could be related to the swelling process and erosion of beads where the degradation of chitosan-coated alginate matrix as the wall materials of the beads has begun. At 240 min of intestinal digestion, micrographs of hydrogel beads (Figures 5m,n) with severe cleavages and uneven surfaces could be observed in the later stage. As shown in Figure 6, the fluorescent micrographs showed a similar microstructure of beads after gastric digestion without any substantial difference. However, the micrographs of beads displayed significant changes at different time points of intestinal digestion. The original compact surface has gradually transformed into a loose structure, indicating the loss of lipid phase from the beads. These results suggest that the digestion could start from the surface toward the center of the beads (48), causing the release of immobilized Pickering emulsion within the beads into the external medium.

### Release of Thymoquinone From Alginate-Chitosan Beads Immobilized Thymoquinone-Loaded Pickering Emulsion

**Figure 7:**
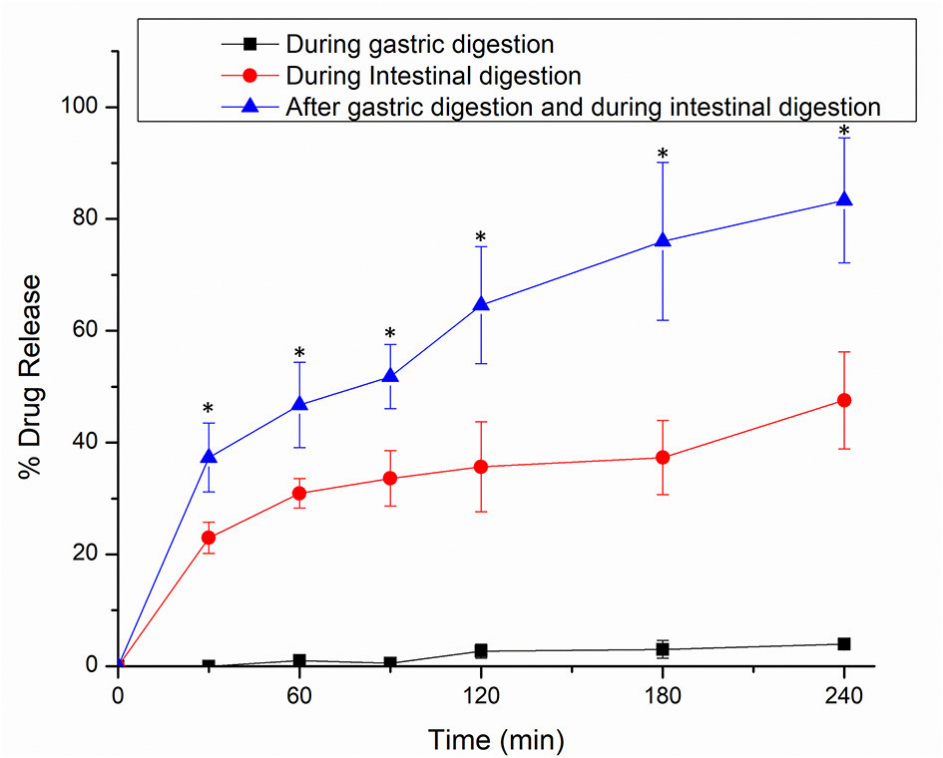
*In vitro* release profiles of thymoquinone from alginate-chitosan hydrogel beads immobilized Pickering emulsion in SGF, SIF, and 2 h in SGF followed by SIF. Error bars represent the standard deviation of three replicates. ^*^Marks significant differences (*p* < 0.05). A comparison was made between 2 h in SGF followed by SIF and SGF/SIF.

The release of encapsulated thymoquinone (TQ) from the hydrogel beads was achieved during gastrointestinal digestion. The TQ release profiles from Alg-Chi-TPE hydrogel beads in three different digestion systems [SGF, SIF, and SIF after pre-soaked in SGF for 2 h (SGF/SIF)] are presented in Figure 7. The release curves in SIF and SGF/SIF media showed a burst release in the initial stage (0–30min) and then a slow release in the following stage (30–240 min). In the first 30min, hydrogel beads in SGF/SIF medium demonstrated a faster release of TQ than in the SIF medium (37 vs. 23%, *p* < 0.05), while TQ releases in the SGF medium is undetected. Only about 4% of TQ was detected after the end of gastric digestion (240 min). The total TQ release in SIF medium and SGF/SIF medium was 48 and 83%, respectively (*p* < 0.05), showing the extensive release of TQ when the hydrogel beads were pre-soaked in the SGF medium before introducing to the SIF medium. The introduction of intestinal fluid at an alkaline pH and the extensive water uptake properties of the hydrogel beads could account for the initial rapid release (11). In addition, the presence of lipase in SIF could initiate the lipolysis process, breaking down lipid droplets into triacylglycerol molecules, mixed micelles, non-digested fat droplets, or smaller fractions of free fatty acids (49, 50), thus releasing TQ in short-chain fatty acids that could be absorbed in the small intestine.

By comparing the TQ release in the SIF medium alone and SGF/SIF medium, it can be denoted that two possible mechanisms regulate the bioactive release out of the hydrogel beads: the swelling process and the diffusion process (31). When the hydrogel beads were soaked in the SGF medium, a syneresis process occurs, resulting in the shrinking of beads. These shrunk beads with lesser volume were later transferred into the SIF medium to initiate intestinal digestion. Following the changes in different pH, the external gelation network structure of hydrogel beads was affected, producing more enormous pores/cleavages, accelerating the swelling process. Then, the encapsulated TQ can be released by diffusion process through the increasingly large openings. During the later stage (60–240 min), the rate of swelling of the beads decreased, and the diffusion process determines the amount of bioactive release.

The results were analyzed using the Peppas model to distinguish the release mechanisms of TQ from the alginate beads. For hydrogel beads, the diffusional exponent (n) specifies the mechanism of release. The calculation of the n value was measured up to the initial 60% release of the bioactive. If the values of n are equal or <0.43, the release is associated with Fickian diffusion. If n values are within 0.43 to 0.85, the release is indicated with Fickian diffusion and Case-II transport (caused by the swelling process). If the values of n are more than 0.85, the release is solely contributed by Case-II transport (32, 51). The results shown in Table 3 indicate that the TQ release from Alg-Chi-TPE hydrogel beads in gastric digestion is associated with Case-II transport since the n value is more significant than 0.85. Thus, the swelling of hydrogel beads only controlled the bioactive release in the gastric stage. In contrast, the n values for TQ release in intestinal digestion, with and without gastric treatment, were lesser than 0.43, indicating that the Fickian diffusion governs the release. The n values in both cases are relatively similar, suggesting that the diffusion of bioactive molecules mainly dominates the release mechanism out of the beads as the digestive medium hydrates the beads.

**Table 3:** Diffusional exponent (*n*), correlation coefficient (*R*^2^), and the transport mechanism of thymoquinone release profile.

	***n***	***R*** ^**2**^	**Transport mechanism**
During gastric digestion	1.147	0.947	Case-II transport
During intestinal digestion	0.311	0.991	Fickian diffusion
After gastric digestion and during intestinal digestion	0.298	0.999	Fickian diffusion

## Conclusion

This work demonstrates *in vitro* digestion and hydrophobic thymoquinone (TQ) release from alginate-chitosan hydrogel beads immobilized Pickering emulsion (Alg-Chi-TPE). 2.0% (w/v) alginate hydrogel beads with spherical shape demonstrated the highest stability against structural deformation during the drying process. The presence of chitosan in beads formation improved the wall materials properties, providing a lower swelling degree and a rougher microstructure. Alg-Chi-TPE hydrogel beads demonstrated good stability during gastric digestion, and the release of encapsulated TQ was observed during intestinal digestion. Up to 83% of total TQ was released from the hydrogel beads after 2 h gastric digestion followed by 4 h treatment in the simulated intestinal fluid. The bioactive release mechanisms were incorporated with the Peppas model, which exhibited a Case-II transport caused by the swelling process during gastric digestion and Fickian diffusion during intestinal digestion. This study contributes to a better understanding of the swelling and digestion behaviors of alginate-chitosan hydrogel beads immobilized with food-grade Pickering emulsions to release a hydrophobic bioactive compound. This provides valuable information about its potential application in developing the colloids-based nutraceutical delivery system.

## Data Availability Statement

The original contributions presented in the study are included in the article/supplementary material, further inquiries can be directed to the corresponding author/s.

## Author Contributions

SKW: conceptualization, methodology, investigation, data curation, formal analysis, and original draft writing. DL and JS: writing-review and editing. CHP and SYT: funding acquisition and validation. BHG, SM, TWW, and CHP: resources and review. SYT: supervision and project administration. All authors contributed to the article and approved the submitted version.

## Funding

The authors gratefully express gratitude to all parties which have contributed toward the success of this project, both financially and technically, especially the S&T Innovation 2025 Major Special Programme (Grant No. 2018B10022) and the Ningbo Natural Science Foundation Programme (Grant No. 2018A610069) funded by the Ningbo Science and Technology Bureau, China, as well as the UNNC FoSE Faculty Inspiration Grant, China. The Zhejiang Provincial Department of Science and Technology is also acknowledged for this research under its Provincial Key Laboratory Programme (2020E10018). This project was supported by the Tropical Medicine and Biology Platform, School of Science and Advanced Engineering Platform, School of Engineering, Monash University Malaysia.

## Conflict of Interest

The authors declare that the research was conducted in the absence of any commercial or financial relationships that could be construed as a potential conflict of interest.

## Publisher's Note

All claims expressed in this article are solely those of the authors and do not necessarily represent those of their affiliated organizations, or those of the publisher, the editors and the reviewers. Any product that may be evaluated in this article, or claim that may be made by its manufacturer, is not guaranteed or endorsed by the publisher.
